# Optimizing Modified Activated Carbon Fiber for Organic Pollutant Removal from Reverse Osmosis Concentrate: Response Surface Modeling and Optimization

**DOI:** 10.3390/ma19061186

**Published:** 2026-03-18

**Authors:** Xiaohan Wei, Aili Gao, Ruijia Ma, Yunchang Huang, Chenglin Liu, Jinlong Wang, Lihua Cheng, Xuejun Bi

**Affiliations:** 1 School of Environmental & Municipal Engineering, Qingdao University of Technology, 777 Jialingjiangdong Road, Qingdao 266520, China; 2 The Fourth Construction Co., Ltd. of China Construction Eighth Engineering Division, China Construction Building, 169 Songling Road, Qingdao 266100, China; 3 Qingdao Jinlonghongye Environmental Protection Co., Ltd., 2 Tianbaoshan Road, Qingdao 266510, China

**Keywords:** ROC, Fe-ACF, organic pollutants, RSM, adsorption mechanism

## Abstract

**Highlights:**

- The adsorption of organics in ROC by Fe-ACF is mainly dominated by chemisorption.
- The Langmuir–Freundlich isotherm model provided the best fit to the experimental data.
- RSM analysis confirmed that pH is the dominant factor affecting Fe-ACF’s adsorption.
- The adsorption mechanism involves hydrogen bonding, π–π interactions, surface complexation, among others.

**Abstract:**

Reverse osmosis concentrate (ROC) contains relatively high levels of refractory organic pollutants, posing significant challenges due to its difficult treatment and high environmental risks. Therefore, efficient and convenient removal strategies are essential. In this study, a self-developed iron-modified activated carbon fiber (Fe-ACF) was employed as an adsorbent to remove organic pollutants from ROC. Additionally, response surface methodology (RSM) was applied to model the adsorption process, identify and evaluate key influencing parameters, and optimize operational conditions. The adsorption mechanisms and regeneration stability of Fe-ACF were also investigated. Kinetic analysis revealed that the adsorption process is predominantly governed by chemisorption, with intraparticle diffusion identified as the primary rate-limiting step. Isothermal adsorption studies demonstrated that the Langmuir–Freundlich model best describes the adsorption behavior, yielding a theoretical maximum adsorption capacity of 12.21 ± 0.80 mg/g. Thermodynamic analysis confirmed that the adsorption process is spontaneous, endothermic, and driven by an increase in entropy. The RSM optimization identified pH as the dominant factor. The optimal adsorption conditions were a pH of 4.18, a temperature of 34.63 °C, a stirring speed of 547.91 rpm, and an adsorbent dosage of 1.55 g/L. The adsorption mechanism involves hydrogen bonding, π–π interactions, surface complexation, and electrostatic forces. Fe-ACF exhibits competitive regeneration stability and structural integrity. In summary, Fe-ACF demonstrates significant potential as a treatment material for ROC.

## 1. Introduction

The escalating scarcity of freshwater resources has become a critical global ecological and environmental issue. Wastewater reclamation offers a sustainable approach by enabling resource recovery to alleviate freshwater demand [1,2]. Reverse osmosis (RO), a highly efficient membrane separation technology, has been extensively applied in seawater desalination, industrial wastewater treatment, and municipal wastewater reuse [3,4]. However, RO processes generate reverse osmosis concentrate (ROC), a high-salinity waste stream containing residual pollutants. The presence of refractory organic compounds, such as humic substances and emerging pollutants, renders ROC treatment technically challenging and economically burdensome while also posing significant environmental risks [5,6]. Consequently, developing efficient, cost-effective, and sustainable treatment strategies for ROC has emerged as a critical priority in water reclamation.

Activated carbon fibers (ACFs), an advanced class of carbonaceous adsorbents, have been widely used to remove organic pollutants due to their exceptionally high specific surface area, well-developed pore structure, superior adsorption capacity, and excellent regeneration performance [7]. Compared to conventional activated carbon, ACFs offer significant advantages in treating low-concentration organic contaminants and in rapid dynamic adsorption applications [8,9]. Previous studies have demonstrated that functional modifications of ACFs—such as oxidative treatments or metal loading (e.g., iron, copper, magnesium)—can effectively modulate surface activity and hydrophilicity, thereby enhancing their adsorption performance toward structurally complex organic pollutants [10,11,12,13]. In our previous foundational work, we developed and optimized a synthesis protocol for iron-modified activated carbon fiber (Fe-ACF) and confirmed that the introduction of Fe_3_O_4_/Fe_2_O_3_ composite oxides significantly increased the mesopore volume and surface chemisorption sites of the carbon matrix [14]. This prior study systematically demonstrated that Fe-ACF exhibited markedly superior removal performance for target organic pollutants compared to pristine ACF. However, to further promote its practical application, a systematic investigation of its key operational parameters is still required, including the long-term operational stability of Fe-ACF, its thermodynamic adsorption capacity in complex water matrices, and its lifespan in engineering application. Moreover, conventional single-factor experimental approaches are often labor-intensive and may compromise the accuracy of results by neglecting interaction effects among variables. Response surface methodology (RSM) is a robust statistical and mathematical modeling technique that enables process optimization with fewer experiments. It also facilitates the development of predictive models that describe the relationships between multiple variables and response outcomes. RSM offers significant advantages in optimizing multi-parameter systems [15,16].

This study employs the well-optimized Fe-ACF material from our previous work to systematically investigate its applicable boundary conditions in high-salinity ROC. In contrast to our previous work, which focused on single-factor optimization of material synthesis conditions, this study evaluates the adsorption performance of Fe-ACF for removing organic pollutants from ROC and optimizes key process parameters using RSM. Furthermore, the adsorption mechanisms were investigated under high-salinity, complex water conditions, specifically elucidating how Fe-mediated chemisorption overcomes the electrostatic shielding inherent in ROC. The regeneration stability and recyclability of the adsorbent were also assessed. Through the integration of laboratory-scale adsorption experiments and model-based analysis, this study demonstrated the applicability and technical advantages of Fe-ACF in treating complex water matrices, offering a viable and environmentally friendly solution for the management of ROC. In addition, this work provides both theoretical insights and technical guidance for the development and optimization of ROC treatment technologies, thereby contributing to the sustainable recovery and reuse of wastewater resources.

## 2. Materials and Methods

### 2.1. Test Water and Adsorbent Materials

The test raw water was ROC, obtained from the secondary reverse osmosis unit of a water reclamation facility located in Qingdao, China. This facility treats secondary effluent from a municipal wastewater treatment plant through a sequential process involving coagulation-sedimentation pretreatment, ultrafiltration, and a two-stage reverse osmosis system to produce high-quality reclaimed water, with a designed treatment capacity of 20,000 m^3^/d. Key water quality parameters of the raw ROC are summarized in Tables S1 and S2 (Supplementary Materials).

The adsorbent used in this study was Fe-ACF, selected based on our previous study [14]. According to the reported characterization, the Fe-ACF exhibited a well-developed porous structure with a specific surface area of 1350.21 m^2^/g and an effective iron loading of 4.57 ± 0.36 wt%. Briefly, the preparation procedure was as follows: pristine ACF was immersed in a 10 wt% Fe(NO_3_)_3_ solution and subjected to ultrasonic-assisted impregnation for 1 h, followed by a high-temperature activation process.

### 2.2. Adsorption Experiment

Batch adsorption experiments were conducted in a 1 L reactor containing ROC. Single-factor adsorption experiments were performed under the following conditions: the solution pH was adjusted within the range of 2 to 10 using 0.5 mol/L NaOH and 0.1 mol/L HCl; the reaction temperature was maintained between 5 and 35 °C using a thermostatic incubator; mass transfer was controlled by varying the stirring speed of a magnetic stirrer (400–900 rpm); and the dosage of Fe-ACF was adjusted from 1 to 5 g/L. During the adsorption process, the concentration of dissolved organic carbon (DOC) in the ROC was monitored, and the equilibrium adsorption capacity of Fe-ACF under various conditions was calculated using Equation (1).

(1) $$Q_{e}\left(mg/g\right)=\frac{{(C}_{0}-C_{e})\times V}{m}$$

where *Q_e_* is the equilibrium adsorption capacity of Fe-ACF (mg/g); *C*_0_ and *C_e_* represent the initial and equilibrium concentrations of DOC in the ROC, respectively (mg/L); *V* is the volume of ROC used in the adsorption experiment (L); and *m* is the mass of Fe-ACF applied (g).

To investigate the adsorption kinetics of Fe-ACF toward organic pollutants in ROC, the pseudo-first-order (PFO) (Equation (2)), pseudo-second-order (PSO) (Equation (3)), and intraparticle diffusion (Equation (4)) models were applied for kinetic fitting and analysis [17].

(2) $$Q_{t}=Q_{e}(1-e^{-k_{1}t})$$

(3) $$Q_{t}=\frac{k_{2}Q_{e}^{2}t}{{1+k}_{2}Q^{e}t}$$

(4) $$Q_{t}=k_{3}t^{\frac{1}{2}}+C$$

where k1, k2, and k3 represent the adsorption rate constants for the pseudo-first-order, pseudo-second-order, and intraparticle diffusion models, respectively; Qt denotes the adsorption capacity of Fe-ACF for organic pollutants at time t (mg/g); t is the adsorption time (min); and C is a constant related to the thickness of the boundary layer.

ROC was diluted to achieve DOC concentrations of 15, 20, 25, 30, and 35 mg/L, respectively. Sodium sulfate and sodium chloride were added to the diluted samples to adjust the sulfate and chloride ion concentrations to levels consistent with those in the original ROC. Subsequently, batch adsorption experiments were conducted to determine the equilibrium adsorption capacity of Fe-ACF at different initial DOC concentrations for adsorption isotherm modeling. The selected isotherm models were the Temkin isotherm (Equation (5)) and the Langmuir–Freundlich isotherm (Equation (6)) [17,18].

(5) $$Q_{e}=Bln(A_{T})+Bln(C_{e})$$

(6) $$Q_{e}=\frac{Q_{m}{\left(bC_{e}\right)}^{n}}{1+{\left(bC_{e}\right)}^{n}}$$

where *A_T_* is the Temkin isotherm equilibrium binding constant (L/mg); *B* is a constant related to the heat of adsorption (mg/g); *Q_m_* is the maximum adsorption capacity of Fe-ACF (mg/g); *b* is the adsorption equilibrium constant (L/mg); *n* is the Freundlich constant.

According to the procedure for the dynamic adsorption experiment, complete the adsorption of pollutants in ROC using the modified ACF material at 288 K (15 °C), 298 K (25 °C), and 308 K (35 °C), respectively. Calculate the thermodynamic adsorption parameters using the following equations (Equations (7)–(9)) [17].

(7) $$K_{d}=\frac{Q_{e}}{C_{e}}$$

(8) $$∆G^{0}=-RTlnK_{e}$$

(9) $$∆G^{0}=∆H^{0}-T∆S^{0}$$

where Kd is the dimensional distribution coefficient (L/g); Ke is the thermodynamic equilibrium constant, equal to 1000 *K*_d_; R is the gas constant (8.314 J/(mol·K)); T is the ambient temperature (K); ΔG0 is the standard Gibbs free energy change (kJ/mol); ΔH0 is the standard enthalpy change, kJ/mol; ΔS0 is the standard entropy change (J/(mol·K)).

### 2.3. Response Surface Experiment

In this study, response surface methodology (RSM) was employed to model and optimize the adsorption conditions influencing the removal of organic pollutants from ROC using modified Fe-ACF. The factors investigated included pH (Factor A), temperature (Factor B), stirring speed (Factor C), and adsorbent dosage (Factor D). Based on preliminary single-factor experiments (Supplementary Figure S1), the center point levels for each factor were set as follows: pH = 4, temperature = 30 °C, stirring speed = 500 rpm, and adsorbent dosage = 2 g/L. A four-factor, three-level Box–Behnken design (BBD) was implemented using Design-Expert 13 software to conduct the experimental design and statistical analysis, with the adsorption capacity of Fe-ACF as the response variable. The factor levels used in the response surface experiments are summarized in Table 1, and the specific experimental design matrix and corresponding results are presented in Table 2.

The relationship between the response variable and the influencing factors was described using a second-order polynomial model (Equation (10)). Analysis of variance (ANOVA) was employed to evaluate the significance of the model for the adsorption of organic pollutants in ROC by Fe-ACF, while residual analysis was conducted to assess the model’s goodness of fit. Furthermore, interaction effects among the adsorption variables were analyzed, and the statistical significance of each model term was determined based on its corresponding F-values and *p*-values.

(10) $$Y=\beta_{0}+\sum_{i=1}^{k}\beta_{i}X_{i}+\sum_{i=1}^{k}\beta_{ii}X_{i}^{2}+\sum_{i=1}^{k}\beta_{ij}X_{i}X_{j}+\varepsilon$$

where Y represents the response variable (adsorption capacity of DOC, mg/g); β0 is the constant term; βi, βii, and βij are the coefficients of the linear, quadratic, and interaction terms, respectively; Xi and Xj are the coded independent variables; and ε denotes the random error.

### 2.4. Regeneration Experiment

The regeneration of Fe-ACF was performed using a solvent-based method. Adsorption-saturated Fe-ACF was first rinsed repeatedly with deionized water to remove surface residues. Then, the material was immersed in a mixed solution of 0.01 mol/L NaOH and 95% ethanol in a 1:1 volume ratio and shaken at room temperature for 30 min. This regeneration step was repeated five times. Finally, the regenerated Fe-ACF was thoroughly rinsed with deionized water and dried in an oven at 85 °C for 24 h, and subsequently sealed and stored for further use.

### 2.5. Characteristic Methods

The concentration of DOC was measured using a total organic carbon analyzer (TOC-L, Shimadzu, Kyoto, Japan). The surface morphology and elemental distribution of Fe-ACF were characterized using scanning electron microscopy (SEM; Sigma 300, Carl Zeiss, Jena, Germany). The specific surface area and pore size distribution were determined using a surface area and porosity analyzer (SSA-4000, Bjbuilder, Beijing, China). The elemental composition and chemical valence states were analyzed by X-ray photoelectron spectroscopy (XPS; K-Alpha, Thermo Scientific, Waltham, MA, USA). Functional groups were identified using a Fourier transform infrared (FTIR) spectrometer (Frontier, PerkinElmer, Waltham, MA, USA) over the spectral range of 4000–400 cm^−1^.

## 3. Results and Discussion

### 3.1. Adsorption Performance of Fe-ACF

**Figure 1:**
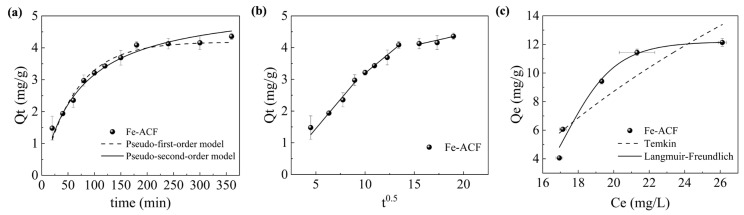
Kinetics fitting curves of (**a**) the pseudo-first-order and pseudo-second-order kinetic models, (**b**) the intraparticle diffusion model, and (**c**) adsorption isotherm fitting: Temkin and Langmuir–Freundlich models for the adsorption of organic pollutants by Fe-ACF.

To evaluate the adsorption performance of Fe-ACF for organic pollutants in ROC, adsorption kinetics experiments were conducted (Figure 1a). At a dosage of 0.77 ± 0.03 g/L, Fe-ACF exhibited excellent adsorption efficiency toward organic pollutants. The adsorption amount reached 93.80% of the saturation adsorption capacity after 3 h and achieved adsorption equilibrium within 6 h. To further elucidate the adsorption mechanism, the kinetic data were fitted using the PFO, PSO, and intraparticle diffusion models. As shown in Supplementary Table S3, both the PFO and PSO models exhibited strong correlations; however, the PSO model demonstrated a higher coefficient of determination (R^2^) and a lower chi-squared (χ^2^), suggesting a better fit. However, as noted in the literature, a superior fit of the PSO model does not constitute definitive evidence of a chemisorption-dominated mechanism [19]. Therefore, the adsorption kinetics were further analyzed using diffusion models.

The fitting results of the intraparticle diffusion model are presented in Figure 1b, with the associated parameters summarized in Table S4 (Supplementary Materials). As illustrated in Figure 1b, the adsorption of organic pollutants from ROC onto Fe-ACF can be divided into three stages. The first stage corresponds to the migration of organic pollutants to the external surface of the Fe-ACF via external diffusion. The second stage involves diffusion within the pores of Fe-ACF, followed by the third stage, which represents the adsorption equilibrium phase. Notably, the fitted lines for all three stages do not intercept the origin, indicating that intraparticle diffusion is not the sole rate-limiting step in the adsorption process [20]. Supplementary Table S4 further reveals that the rate constant for external diffusion (K_3,1_) is higher than that for intraparticle diffusion (K_3,2_), indicating that migration from ROC to the Fe-ACF surface occurs rapidly, whereas intraparticle diffusion constitutes the primary rate-limiting step during adsorption [21].

To investigate the adsorption behavior of Fe-ACF toward organic pollutants in ROC, adsorption isotherm data within an initial DOC concentration range of 15–35 mg/L were fitted using the Temkin and Langmuir–Freundlich models (Figure 1c). As the initial DOC concentration increased, the adsorption capacity of Fe-ACF exhibited an upward trend, reaching a maximum of 12.13 ± 0.27 mg/g. The isotherm fitting parameters are summarized in Table S5 (Supplementary Materials). The Langmuir–Freundlich model provided a superior fit (R^2^ = 0.97), indicating that adsorption occurs on a heterogeneous surface [17]. The surface heterogeneity was caused by the iron loaded onto the ACF surface, which enhanced the adsorption capacity of Fe-ACF [22]. Furthermore, based on the Langmuir–Freundlich model, the theoretical maximum adsorption capacity of Fe-ACF for organic pollutants in ROC was calculated to be 12.21 ± 0.80 mg/g, exceeding that of conventional carbon materials in ROC (Table 3). This enhanced performance is primarily attributed to the well-developed pore structure and surface chemical properties of Fe-ACF, which not only provide abundant diffusion pathways to accelerate adsorption kinetics but also offer increased active sites to achieve a high adsorption capacity [23]. Therefore, these diffusion analyses reveal that the overall adsorption mechanism is a complex mass-transfer process governed jointly by external boundary layer diffusion and intraparticle diffusion, rather than being driven solely by simple surface chemisorption.

To further elucidate the thermodynamic characteristics of the Fe-ACF adsorption of organic pollutants in ROC, thermodynamic analysis was conducted based on adsorption data at 288, 298, and 308 K (Figure S2). The calculated thermodynamic parameters are presented in Table 4. The positive value of the standard enthalpy change (Δ*H*^0^) indicates that the adsorption behavior of Fe-ACF in ROC is an endothermic process. Moreover, the magnitude of Δ*H*^0^, falling between typical physisorption (<20 kJ/mol) and chemisorption (>80 kJ/mol), suggests that the process is governed by a synergistic physicochemical mechanism. The calculated standard Gibbs free energy changes (Δ*G*^0^) were all negative, decreasing from −12.86 to 16.53 kJ/mol as the temperature increased from 288 to 308 K. This indicates that the adsorption of Fe-ACF in ROC is a thermodynamic spontaneous process under standard conditions, and the decreasing trend suggests that higher temperatures make the process progressively more thermodynamically favorable. Furthermore, the positive standard entropy change (Δ*S*^0^) indicates an increase in randomness at the solid–liquid interface during Fe-ACF adsorption, suggesting that the adsorption of organic pollutants by Fe-ACF is primarily an entropy-driven process [24].

**Table 3:** Comparison of maximum adsorption capacities of different adsorbents for organic pollutants in ROC or similar matrices.

Adsorbent	Adsorbate	*Q*_max_ (mg/g)	Reference
GAC	ROC	3.00	Jamil et al. [25]
PAC	surface water	3.85	Sangkarak et al. [26]
biochar	river water	6.40	Lee et al. [27]
Fe-ACF	ROC	12.21	This study

Notes: *Q*_max_ indicates the maximum adsorption capacity, GAC indicates the granular activated carbon, PAC indicates the powdered activated carbon, and ROC indicates the reverse osmosis concentrate.

**Table 4:** Thermodynamic parameters for the adsorption of organic pollutants by Fe-ACF.

T (K)	lnK	ΔG^0^ (kJ/mol)	ΔH^0^ (kJ/mol)	ΔS^0^ (J/mol/K)
288	5.38	−12.89	39.35	180.85
298	5.74	−14.21		
308	6.46	−16.53		

### 3.2. RSM Model Analysis and Optimization of Fe-ACF Adsorption Efficiency

Based on 29 randomly arranged experimental runs listed in Table 2 (comprising 24 edge midpoints and 5 center points), a quadratic polynomial model was developed to describe the effects of four process variables—pH (A), temperature (B), stirring speed (C), and adsorbent dosage (D)—on the Fe-ACF adsorption capacity (y), as expressed in Equation (11):

(11) $$y=-19.906+6.295A-0.150B+0.032C+5.333D-0.034AB+0.004AC+0.350AD+0.001BC+0.018BD-0.015CD-0.971A^{2}-0.005B^{2}-0.00005C^{2}-0.129D^{2}$$

The statistical validation and adequacy of the developed model were assessed using analysis of variance (ANOVA) (Table 5). The relatively high model F-value of 10.82 indicates that the model is statistically significant, with the regression between Fe-ACF adsorption capacity and the influencing factors demonstrating high significance (*p* < 0.0001). Moreover, factors A (pH), B (temperature), D (adsorbent dosage), interaction terms BC and CD, as well as the quadratic term A2, were identified as significant contributors to the model (*p* < 0.05). Meanwhile, the non-significant lack-of-fit term adequately fits the experimental data.

To further evaluate the reliability and predictive capability of the model, the fit statistics are summarized in Table 6. The coefficient of variation (C.V.) was 5.70%; a C.V. value well below 10% indicates experimental reproducibility and data reliability. The coefficient of determination (R^2^) was 0.9154, while the difference between the adjusted coefficient of determination (R^2^_adj_) and the predicted coefficient of determination (R^2^_pred_) was 0.2884, which does not meet the commonly accepted standards (generally, R^2^ > 0.95 and a difference between R^2^_adj_ and R^2^_pred_ of less than 0.2 are desired). This discrepancy is primarily due to the actual ROC matrix being more complex than the idealized single-solute adsorption system, exhibiting multi-component characteristics. The presence of various refractory organic compounds and high salinity introduces strong competitive adsorption kinetics and unpredictable nonlinear interactions, limiting the predictive capability of idealized mathematical models. The adequate precision, which assesses the signal-to-noise ratio, was 13.0683, exceeding the desirable threshold of 4.0 and demonstrating a signal to reliably navigate the design space. Considering the low C.V. and high adequate precision, the established quadratic model remains statistically robust and practically realistic for describing the adsorption process of actual ROC.

**Figure 2:**
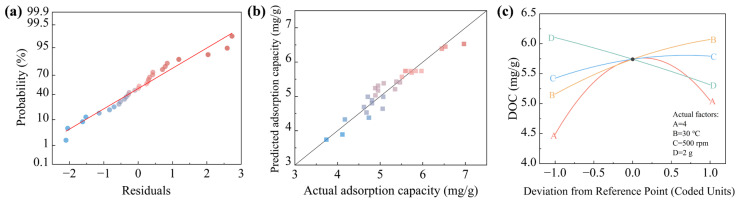
Residual normal probability distribution diagram of the adsorption amount of organic pollutants in ROC by Fe-ACF (**a**), comparison of actual and predicted values (**b**), and factor disturbance effect curve (**c**).

Residual analysis and comparison between the predicted and experimental adsorption capacities were conducted to evaluate the model fit (Figure 2). As shown in Figure 2a, the residuals were approximately aligned along the regular probability line, indicating that they followed a normal distribution. Furthermore, the predicted adsorption capacities exhibited strong agreement with the experimental values (Figure 2b), as most data points lay close to the 1:1 reference line, confirming the high predictive accuracy, robustness, and reliability of the model. Figure 2c displays the perturbation curves of four process parameters—pH (A), temperature (B), stirring speed (C), and adsorbent dosage (D)—on the adsorption capacity of Fe-ACF. The slope of each curve reflects the sensitivity of the response to that specific variable. Among these, pH (A) exhibited the highest sensitivity, as deviations from its central value resulted in pronounced changes in adsorption performance. The relative sensitivities of the remaining factors followed the order: B > D > C (temperature > adsorbent dosage > stirring speed). This trend can be attributed to the fact that the adsorption process is primarily driven by the affinity between the adsorbent and the adsorbate. The pH plays a pivotal role in modulating both the physicochemical properties of organic pollutants and the surface characteristics of the adsorbent, thereby directly influencing the adsorption affinity [28].

**Figure 3:**
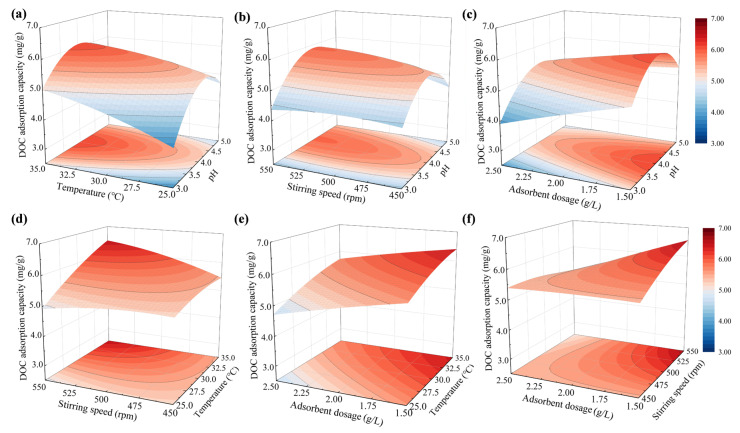
Response surface plots of the interactive effects of different variables on the adsorption of organic pollutants by Fe-ACF. (**a**) pH and temperature, (**b**) pH and stirring speed, (**c**) pH and adsorbent dosage, (**d**) temperature and stirring speed, (**e**) temperature and adsorbent dosage, (**f**) stirring speed and adsorbent dosage.

The interaction effects of pH, temperature, stirring speed, and adsorbent dosage on the adsorption capacity of Fe-ACF were investigated using three-dimensional response surface plots and corresponding contour plots (Figure 3). Among these variables, pH emerged as the dominant factor, exerting the most pronounced influence across all interaction terms. In the pH–temperature (Figure 3a), pH–stirring speed (Figure 3b), and pH–adsorbent dosage (Figure 3c) plots, the response surfaces exhibited distinct peaks near the pH center point, accompanied by steep gradients, indicating strong interactive effects between pH and the other variables. Notably, pH had a significant impact on adsorption capacity. As the pH increased, the DOC adsorption capacity first increased and then declined, reaching a maximum of approximately 6.13 mg/g at a pH of 4.03. This trend can be attributed to two primary factors. First, the organic pollutants in ROC are predominantly negatively charged humic substances. According to previous characterization data of Fe-ACF, its point of zero charge (pH_pzc_) is approximately 4.91 [14]. Therefore, under acidic conditions where the solution pH is below the pH_pzc_, the surface charge of Fe-ACF shifts from negative to positive, thereby enhancing electrostatic attraction toward these anionic species [29]. Second, a low pH reduces the degree of ionization of organic molecules and enhances their hydrophobicity, which facilitates adsorption onto the Fe-ACF surface [28]. In contrast, temperature exhibited a moderate influence within the tested range. A moderate increase in temperature led to a slight increase in DOC adsorption capacity. This effect occurs because increasing the temperature can enhance DOC adsorption by improving the diffusion kinetics and promoting a thermodynamically favorable endothermic adsorption process, as previously confirmed by our thermodynamic analysis. This is evidenced by the gentle gradients observed in the temperature–stirring speed (Figure 3d) and temperature–adsorbent dosage (Figure 3e) response surfaces. Increased stirring can enhance mass transfer, and selecting an appropriate stirring speed is crucial for balancing adsorption efficiency and preserving the structural integrity of the adsorbent. However, within the studied range, variations in stirring speed only had a minor effect compared to pH and adsorbent dosage, suggesting that the adsorption process is primarily governed by surface interaction mechanisms (Figure 3b,d,f). In contrast, adsorbent dosage exerted a substantial and direct effect across all interaction plots, underscoring the critical role of available surface area and active binding sites in enhancing DOC adsorption capacity (Figure 3c,e,f). Collectively, these findings indicate that DOC adsorption onto Fe-ACF is primarily governed by pH and adsorbent dosage, while temperature and stirring speed serve as auxiliary factors that have a slight impact on overall performance.

Based on the fitted response surface model, the optimal adsorption conditions were determined as follows: pH 4.18, temperature 34.63 °C, stirring speed 547.91 rpm, and adsorbent dosage 1.55 g/L. Under these optimized conditions, Fe-ACF achieved a predicted DOC adsorption capacity of 7.00 mg/g. Validation experiments conducted in triplicate under these conditions yielded an average DOC removal of 6.61 ± 0.04 mg/g. Although the model slightly overestimated the adsorption performance by approximately 5.57%, its predictive accuracy remained acceptable, confirming the model’s reliability in simulating the adsorption of organic matter in ROC by Fe-ACF. It is important to maintain the response within a practical and acceptable range during the optimization process to ensure the feasibility of the resulting solutions. Accordingly, control parameters should be adjusted within reasonable operational limits to reflect the real-world conditions accurately.

### 3.3. The Adsorption Mechanism of Fe-ACF for Organic Pollutants in ROC

**Figure 4:**
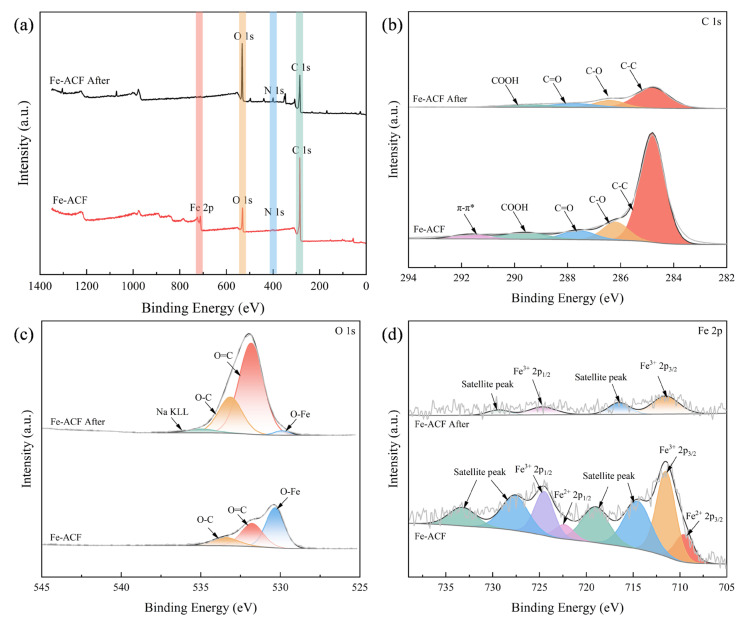
XPS spectra of Fe-ACF before and after pollutant adsorption. (**a**) Wide-scan survey spectrum; high-resolution spectra of (**b**) C 1s, (**c**) O 1s, and (**d**) Fe 2p.

To investigate the adsorption mechanism of Fe-ACF toward organic pollutants in ROC, XPS was employed to analyze the elemental composition and chemical states of Fe-ACF before and after adsorption (Figure 4). Compared to pristine Fe-ACF, the full-scan XPS spectrum of Fe-ACF after adsorption exhibited a pronounced decrease in the peak intensities corresponding to C 1s, O 1s, and Fe 2p, along with the emergence of new peaks at several binding energy positions (Figure 4a). These changes indicate that organic pollutants from ROC were successfully adsorbed onto the Fe-ACF surface, accompanied by significant chemical interactions among the carbon, oxygen, and iron species during the adsorption process.

To further elucidate the surface interactions involved in the adsorption process, peak deconvolution was performed on the high-resolution XPS spectra of C 1s, O 1s, and Fe 2p. As shown in Figure 4b, the C 1s spectrum of Fe-ACF after adsorption revealed characteristic peaks corresponding to C–C (284.80 eV), C–O (286.40 eV), C=O (287.75 eV), and COOH (289.54 eV) [10]. Compared to pristine Fe-ACF, the intensities of all four peaks decreased, and the π–π* satellite peak disappeared after adsorption, indicating a significant alteration in the carbon chemical environment and a π-conjugated system on the Fe-ACF surface. This change can be primarily attributed to the coverage of organic compounds from ROC on the Fe-ACF surface, which attenuates the original signals of carbon-based functional groups. The pronounced enhancement of the alkane C–H deformation vibration band at 1380–1385 cm^−1^ in the FTIR spectrum [30] further corroborates the presence of adsorbed organic species (Supplementary Figure S3). In addition, hydrogen bonding and π–π interactions between surface functional groups on Fe-ACF and organic compounds in ROC may induce transformations of the original carbon functionalities. Furthermore, charge disturbances on the Fe-ACF surface during adsorption may disrupt the delocalized π-electron system. Both effects can contribute to the disappearance of the π–π* satellite peak [31,32]. These observations support the occurrence of strong chemisorption between Fe-ACF and the organic constituents in ROC, consistent with the chemisorption-dominated mechanism inferred from kinetic analysis.

The high-resolution O 1s spectrum (Figure 4c) shows a marked increase in the peak intensities corresponding to the C=O and C–O groups after adsorption, indicating that oxygen-containing organic pollutants in ROC are effectively adsorbed by Fe-ACF. This interpretation is further supported by the FTIR spectrum, which showed a significant increase in the absorption band at 1100 cm^−1^, attributed to C–O stretching vibrations [33] (Supplementary Figure S3). Conversely, the intensity of the Fe–O peak significantly decreased, likely due to coordination interactions between surface iron species and functional groups (e.g., carboxyl groups) in humic substances and other organic compounds present in ROC [34], resulting in the consumption of reactive Fe–O sites. Additionally, the appearance of a distinct Na KLL Auger peak at approximately 534.95 eV suggests that Na^+^ ions in ROC may potentially co-adsorb or interact with the Fe-ACF surface during the adsorption process. The high-resolution Fe 2p spectrum of Fe-ACF after adsorption (Figure 4d) displayed characteristic peaks of Fe^3+^, with binding energies at 711.54 eV and 724.68 eV, corresponding to the Fe 2p_3_/_2_ and Fe 2p_1_/_2_ orbitals of Fe^3+^, respectively [35]. Compared to pristine Fe-ACF, which exhibited characteristic peaks of both Fe^3+^ and Fe^2+^, the intensities of the Fe^3+^ peaks in Fe-ACF after adsorption decreased, while no discernible peaks associated with Fe^2+^ were observed. This suggests that the surface iron species on Fe-ACF underwent transformation during the adsorption process, potentially due to complexation with electron-donating functional groups in humic substances [36], as well as physical surface masking by adsorbed organics and complexation-induced electron transfer. In summary, the adsorption mechanism of Fe-ACF for organic pollutants in ROC primarily involves: (1) hydrogen bonding and π–π interactions between aromatic organics and the carbon matrix; (2) surface complexation between Fe species on Fe-ACF and organic constituents in ROC; and (3) electrostatic interactions between organic constituents and Fe-ACF.

### 3.4. The Regeneration Performance of Fe-ACF

**Figure 5:**
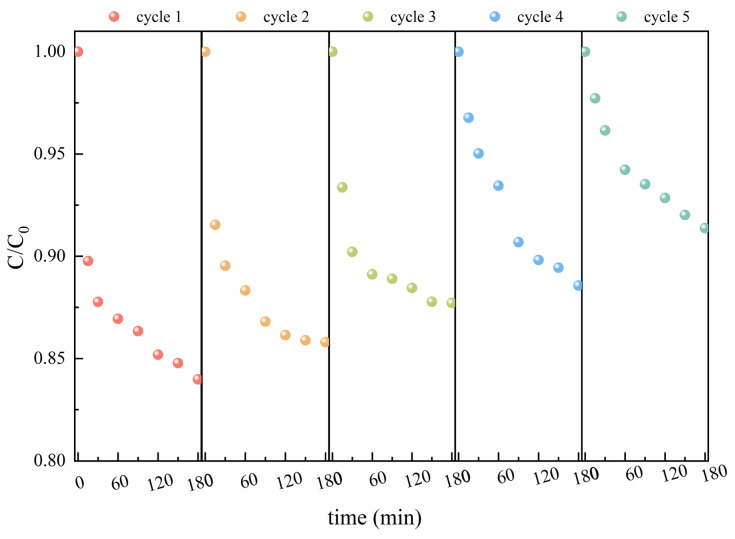
Reusability and regeneration performance of Fe-ACF in ROC for the adsorption of organic pollutants.

To evaluate the reusability and regeneration performance of Fe-ACF, multiple adsorption–regeneration cycles were conducted. As shown in Figure 5, the adsorption capacity of Fe-ACF for organic pollutants in ROC exhibited a gradual decline with increasing regeneration cycles. The initial adsorption capacity of pristine Fe-ACF was 7.86 mg/g. After the first regeneration cycle, the removal efficiency and capacity retention were 88.64% and 82.86%, respectively, of those achieved by the pristine adsorbent. During the second and third cycles, the capacity retentions were 68.93% and 68.46%, respectively. After four adsorption–desorption cycles, the adsorption capacity stabilized at 3.65 mg/g, which remained significantly higher than the initial adsorption capacity of unmodified ACF and conventional GAC (~3.0 mg/g) reported for ROC treatment [25]. BET characterization revealed a 25.73% reduction in specific surface area and a 29.17% decrease in micropore volume after regeneration. This deterioration is primarily attributed to the collapse or merging of micropores during the regeneration process. Notably, the pore size distribution remained favorable, with no significant change in total pore volume, while the mesopore volume increased by 72.00% (Supplementary Figure S4). The decline in adsorption performance can be attributed, in part, to the loss of accessible adsorption sites resulting from the reduced surface area. Additionally, SEM images (Supplementary Figure S5) demonstrated that the fibrous structure of Fe-ACF remained morphologically intact after regeneration. Elemental mapping via EDS analysis confirmed that iron was still uniformly dispersed on the ACF support, with its elemental composition remaining essentially unchanged compared to that of the pristine Fe-ACF. Therefore, Fe-ACF exhibits competitive regeneration and structural stability, highlighting its strong potential for removing organic pollutants from reclaimed water. From a practical application perspective, this acceptable reusability indicates that Fe-ACF can be utilized in semi-continuous engineering systems employing a periodic make-up strategy (i.e., replacing a small fraction of spent carbon with fresh material). This approach significantly reduces the overall material consumption and operational costs compared to single-use applications. For spent Fe-ACF that is no longer suitable for regeneration or reuse, environmentally sound disposal methods—such as secure landfilling or incineration with energy recovery—should be employed to minimize the risk of secondary pollution.

## 4. Conclusions

This study investigated the adsorption behavior, underlying mechanisms, and regeneration performance of modified Fe-ACF with optimized adsorption properties for organic pollutants in ROC. The findings revealed that the adsorption process was primarily governed by chemisorption, with intraparticle diffusion identified as a major, though not exclusive, rate-limiting step. The Langmuir–Freundlich isotherm model provided the best fit to the experimental data, yielding a theoretical maximum adsorption capacity of 12.21 ± 0.80 mg/g. Thermodynamic evaluations demonstrated that the adsorption of organic pollutants onto Fe-ACF is a spontaneous, endothermic, and entropy-driven process. Among the parameters studied, RSM analysis indicated that pH, temperature, and adsorbent dosage were significant influencing factors, with sensitivity ranked in the following order: pH > temperature > adsorbent dosage > stirring speed. Interaction analysis further confirmed pH as the dominant factor. Model optimization identified the optimal adsorption conditions as pH 4.18, temperature 34.63 °C, stirring speed 547.91 rpm, and adsorbent dosage 1.55 g/L, under which the DOC adsorption capacity reached 7.00 mg/g.

The primary adsorption mechanisms were: (1) hydrogen bonding and π–π interactions between aromatic organics and the carbon matrix; (2) surface complexation between Fe species and organic molecules; and (3) electrostatic interactions. Adsorption–regeneration cycling experiments, combined with BET and SEM characterization, demonstrated that Fe-ACF exhibits excellent reusability and structural integrity, highlighting its potential application for removing organic pollutants from ROC.

## Figures and Tables

**Table 1:** Experimental factors and level ranges in Box–Behnken design for ACF adsorption optimization.

Level	Factor			
	A- pH	B- Temperature (°C)	C- Stirring Speed (rpm)	D- Adsorbent Dosage (g/L)
−1	3	25	450	1.5
0	4	30	500	2
1	5	35	550	2.5

**Table 2:** Response surface experimental design plan and experiment results.

Run	Factor				Response
	pH	Temperature (°C)	Stirring Speed (rpm)	Adsorbent Dosage (g/L)	DOC Adsorption Capacity (mg/g)
1	4	30	500	2	5.72
2	3	30	500	2.5	4.11
3	4	25	450	2	4.95
4	3	30	500	1.5	4.89
5	5	30	500	2.5	4.81
6	4	35	550	2	6.53
7	4	30	500	2	5.83
8	4	30	500	2	5.60
9	5	25	500	2	5.06
10	4	30	550	2.5	5.07
11	4	25	550	2	4.81
12	5	30	550	2	4.95
13	4	30	500	2	5.59
14	4	30	550	1.5	6.97
15	5	30	450	2	4.68
16	3	25	500	2	3.74
17	4	35	450	2	5.38
18	4	25	500	1.5	5.51
19	4	30	450	2.5	5.08
20	5	30	500	1.5	4.88
21	5	35	500	2	5.35
22	4	35	500	1.5	6.45
23	4	25	500	2.5	4.62
24	4	30	450	1.5	5.47
25	3	30	450	2	4.73
26	3	30	550	2	4.17
27	4	30	500	2	5.97
28	4	35	500	2.5	5.74
29	3	35	500	2	4.71

**Table 5:** Analysis of variance of the response surface experiment results.

Source	Sum of Squares	df	Mean Square	*F*-Value	*p*-Value	Significance
Model	13.40	14	0.96	10.82	<0.0001	***
A-pH	0.95	1	0.95	10.71	0.006	**
B-temperature	2.49	1	2.49	28.16	0.000	***
C-stirring speed	0.41	1	0.41	4.60	0.050	-
D-adsorbent dosage	1.86	1	1.86	20.99	0.000	***
AB	0.12	1	0.12	1.32	0.271	-
AC	0.17	1	0.17	1.96	0.183	-
AD	0.12	1	0.12	1.38	0.259	-
BC	0.42	1	0.42	4.70	0.048	*
BD	0.01	1	0.01	0.09	0.763	-
CD	0.57	1	0.57	6.45	0.024	*
A^2^	6.12	1	6.12	69.19	<0.0001	***
B^2^	0.10	1	0.10	1.18	0.296	-
C^2^	0.11	1	0.11	1.29	0.275	-
D^2^	0.01	1	0.01	0.08	0.787	-
Residual	1.24	14	0.09			
Lack of Fit	1.13	10	0.11	4.37	0.084	
Pure Error	0.10	4	0.03			
Cor Total	14.64	28				

Notes: * indicates a significance level of 0.05, ** indicates a significance level of 0.01, and *** indicates a significance level of 0.001.

**Table 6:** The fit statistic results of the Box–Behnken model.

Std. Dev.	C.V. %	R^2^	R^2^_adj_	R^2^_pred_	Adeq Precision
0.30	5.70	0.9154	0.8308	0.5423	13.07

## Data Availability

The original contributions presented in this study are included in the article/Supplementary Material. Further inquiries can be directed to the corresponding author.

## Supplementary Materials

The following supporting information can be downloaded at: https://www.mdpi.com/article/10.3390/ma19061186/s1, Figure S1: Effects of various factors on the adsorption of organic pollutants by Fe-ACF: (**a**) pH, (**b**) temperature, (**c**) stirring speed, and (**d**) adsorbent dosage; Figure S2: Van′t Hoff plot for the adsorption of organic pollutants by Fe-ACF; Figure S3: FTIR spectra of Fe-ACF before and after pollutant adsorption; Figure S4: N_2_ adsorption–desorption isotherm of Fe-ACF and Fe-ACF after regeneration, along with the pore size distribution (inset), and the physical parameters; Figure S5: SEM micrographs (**a**), and EDS mapping of the major elements on the surfaces of Fe-ACF after regeneration (**b**,**c**). Table S1: Basic indicators of ROC of the experiments; Table S2: Inorganic element indicators of ROC of the experiments; Table S3: Fitting results of pseudo-first and pseudo-second kinetic models for Fe-ACF adsorption of organic pollutants; Table S4: Fitting results of the intraparticle diffusion model for Fe-ACF adsorption of organic pollutants; Table S5. Fitting results of adsorption isotherm models for Fe-ACF adsorbing organic pollutants.
