# Supplementary material for: Optimizing Modified Activated Carbon Fiber for Organic Pollutant Removal from Reverse Osmosis Concentrate: Response Surface Modeling and Optimization
Source: Materials (Basel). 2026 Mar 18;19(6):1186. doi: 10.3390/ma19061186 (PMC13027874; doi:10.3390/ma19061186)
Supplement: Supplementary file 1 [file materials-19-01186-s001.zip › materials-4160490-supplementary.pdf]

Appendix A. Supplementary Materials

Optimizing modified activated carbon fiber for organic pollutant removal from reverse osmosis concentrate: response surface modelling and optimization

Xiaohan Wei [a], Aili Gao [a], Ruijia Ma [a], Yunchang Huang [b], Chenglin Liu [b], Jinlong Wang [c], Lihua Cheng [a, *], Xuejun Bi [a]

[a] School of Environmental & Municipal Engineering, Qingdao University of Technology, 777 Jialingjiangdong Road, Qingdao 266520, China

[b] The Fourth Construction Co., Ltd of China Construction Eighth Engineering Division, China Construction Building, 169 Songling Road, Qingdao 266100, China

[c] Qingdao Jinlonghongye Environmental Protection Co. Ltd, 2 Tianbaoshan Road, Qingdao 266510, China

*Corresponding author: Lihua Cheng

Tel: +86 532 85071238

Fax: +86 532 85071238

Email: lihuacheng@qut.edu.cn

Full postal address: 777, Jialingjiangdong Road, 266520 Qingdao, China

Fig. S1

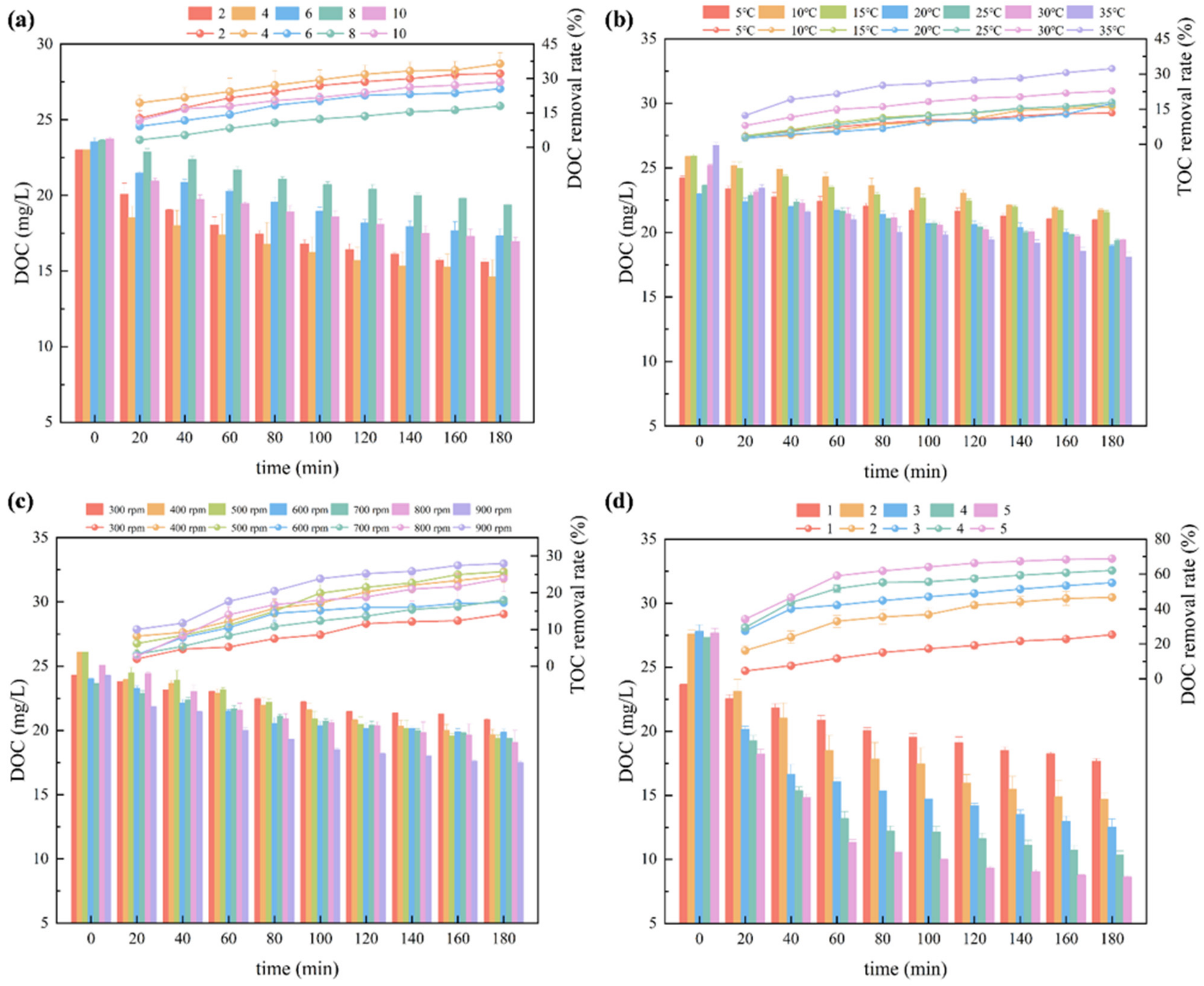

Figure S1. Effects of various factors on the adsorption of organic pollutants by Fe-ACF: (**a**) pH, (**b**) temperature, (**c**) stirring speed, and (**d**) adsorbent dosage.

Fig. S2

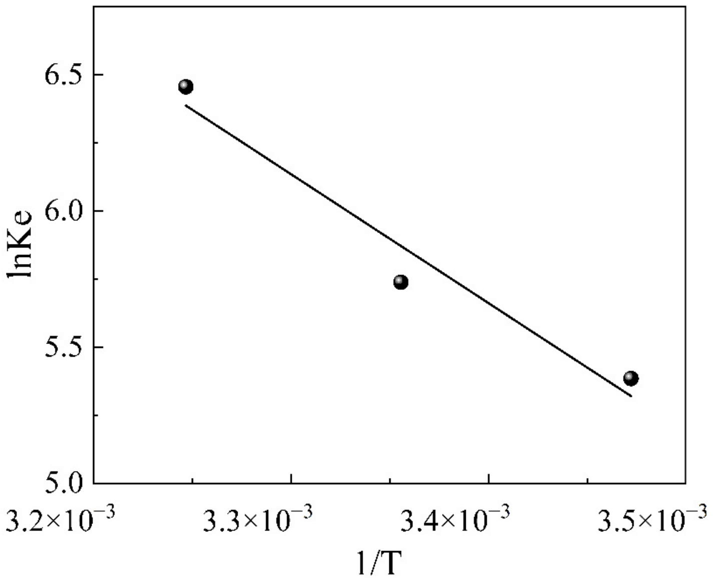

Figure S2. Van't Hoff plot for the adsorption of organic pollutants by Fe-ACF.

Fig. S3

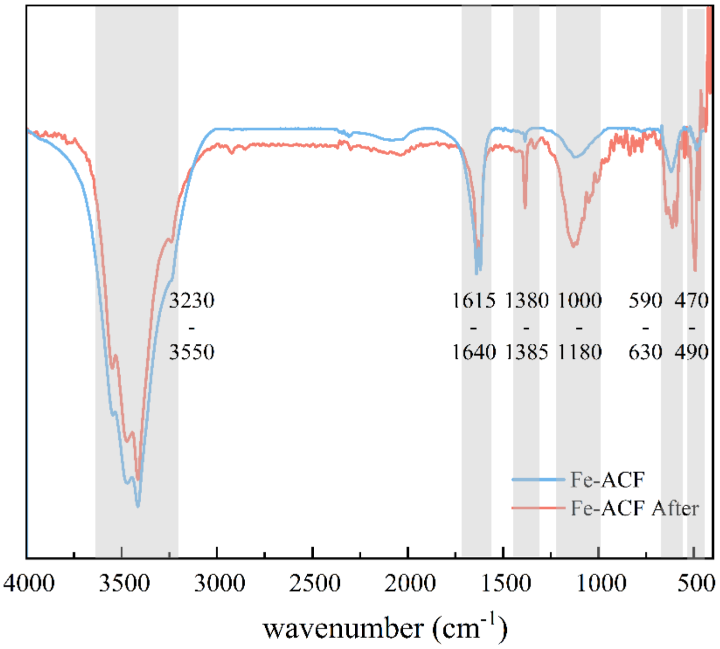

Figure S3. FTIR spectra of Fe-ACF before and after pollutant adsorption.

Fig. S4

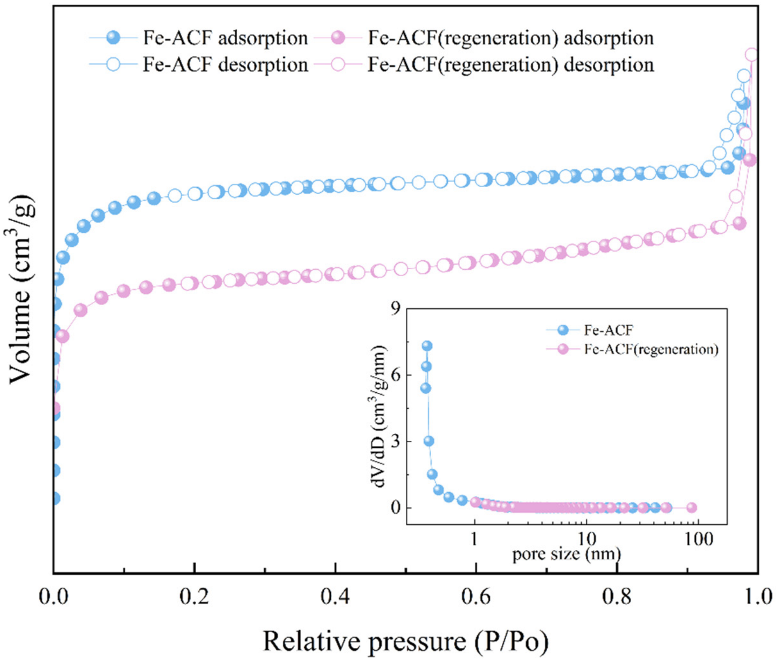

Adsorbent	Fe-ACF	Fe-ACF (regeneration)
BET surface area/ m^2/g	1350.21	1002.76
Total pore volume/ cm^3/g	0.74	0.77
Micropore volume/ cm^3/g	0.48	0.34
Mesopore volume/ cm^3/g	0.25	0.43
Average aperture/ nm	1.09	1.54
Average Mesopore aperture/ nm	5.27	7.26

Pore structure parameters

Figure S4. N_2 adsorption–desorption isotherm of Fe-ACF and Fe-ACF after regeneration, along with the pore size distribution (inset), and the physical parameters.

Fig. S5

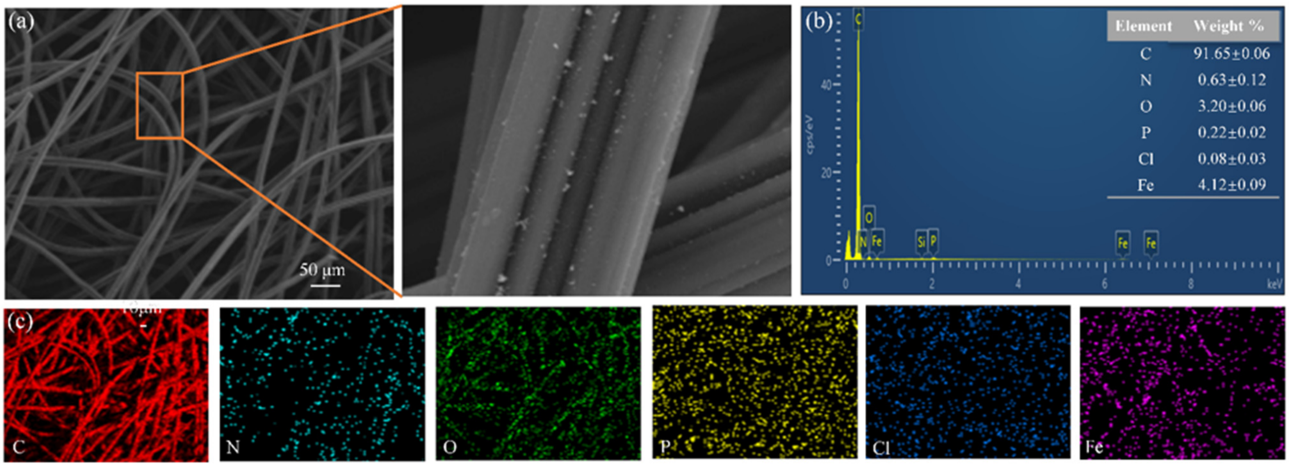

Figure S5. SEM micrographs (**a**), and EDS mapping of the major elements on the surfaces of Fe-ACF after regeneration (**b**, **c**).

Table

Table S1. Basic indicators of ROC of the experiments [1].

Parameter	pH	Salinity (%)	TDS (mg/L)	Conductivity (mS/cm)	DOC (mg/L)	TN (mg/L)	TP (mg/L)
Value	8.42 ± 0.38	0.44 ± 0.05	4.21 ± 0.36	8.36 ± 0.76	33.19 ± 5.54	29.59 ± 3.82	0.33 ± 0.05

Table S2. Inorganic element indicators of ROC of the experiments [1].

Parameter	Concentration	Parameter	Concentration	Parameter	Concentration
F^- (mg/L)	11.72 ± 1.36	Cl^- (mg/L)	2536.55 ± 330.78	SO_4^{2-} (mg/L)	1188.29 ± 95.39
Na (mg/L)	1002.26 ± 8.23	Mg (mg/L)	153.67 ± 5.56	Si (mg/L)	8.93 ± 0.33
K (mg/L)	97.43 ± 2.19	Ca (mg/L)	388.95 ± 5.40	Al (μg/L)	80.78 ± 7.88
Mn (μg/L)	153.86 ± 6.70	Fe (μg/L)	31.05 ± 3.39	Sr (μg/L)	1946.60 ± 60.10
Ba (μg/L)	256.27 ± 24.20				

Table S3. Fitting results of pseudo-first and pseudo-second kinetic models for Fe-ACF adsorption of organic pollutants.

	$Q_{e,exp}$ (mg/g)	PFO				PSO			
		$Q_{e,\,cal}$ (mg/g)	k_1 (/min)	R^2	χ^2	$Q_{e,\,cal}$ (mg/g)	k_2 (g/(mg·min))	R^2	χ^2
Fe-ACF	4.36	4.18	0.0155	0.9946	1.6<br>9	5.43	0.0026	0.9962	1.19

Table S4. Fitting results of the intraparticle diffusion model for Fe-ACF adsorption of organic pollutants.

	External diffusion		Intraparticle diffusion		Equilibrium	
	$K_{3,1}$ (mg/(g min$^{0.5}$)	R^2	$K_{3,2}$ (mg/(g min$^{0.5}$)	R^2	$K_{3,3}$ (mg/(g min$^{0.5}$)	R^2
Fe-ACF	0.3688	0.9813	0.2552	0.9944	0.0700	0.9170

Table S5. Fitting results of adsorption isotherm models for Fe-ACF adsorbing organic pollutants.

	Temkin			Langmuir-Freundlich			
	A_T (L/mg)	B (mg/g)	R^2	Q_m (mg/g)	b (L/mg)	n	R^2
Fe-ACF	0.0822	17.5304	0.8150	12.2106	0.05713	13.1020	0.9740

Reference

[1] X. Wei, C. Wang, T. Zhao, W. Wang, W. Song, L. Cheng, S. Huang, X. Bi, Development of iron-modified activated carbon fiber (Fe-ACF) for organic pollutant removal from reverse osmosis concentrate: Modification conditions optimization and adsorption mechanism, J. Taiwan Inst. Chem. Eng. 182 (2026) 106608. https://doi.org/10.1016/j.jtice.2025.106608.
